# Aging extension and modifications of lipid metabolism in the monogonont rotifer *Brachionus koreanus* under chronic caloric restriction

**DOI:** 10.1038/s41598-018-20108-7

**Published:** 2018-01-29

**Authors:** Min-Chul Lee, Jun Chul Park, Deok-Seo Yoon, Jeonghoon Han, Sujin Kang, Shohei Kamizono, Ae-Son Om, Kyung-Hoon Shin, Atsushi Hagiwara, Jae-Seong Lee

**Affiliations:** 1 Department of Biological Science, College of Science, Sungkyunkwan University, Suwon, 16419 South Korea; 2 Department of Marine Sciences and Convergent Technology, Hanyang University, Ansan, 15588 South Korea; 3 Graduate School of Fisheries and Environmental Sciences, Nagasaki University, Nagasaki, 852-8521 Japan; 4 Department of Food and Nutrition, College of Human Ecology, Hanyang University, Seoul, 04763 South Korea

## Abstract

To examine the interrelationship of aging extension and modification of lipid metabolism under chronic caloric restriction (CCR; reduced concentration of the green algae *Tetraselmis suecica*) in the monogonont rotifer *Brachionus koreanus*, we assessed life cycle parameters, fatty acid composition, and expression of sirtuin and genes related to lipid metabolism. *B*. *koreanus* in the 5% *T*. *suecica* group showed an increased life span but decreased reproduction. Based on this finding, we chose 5% *T*. *suecica* for further experiments and compared the data with those for 100% *T*. *suecica*. Upregulation of sirtuin gene expression was observed under CCR. In addition, despite the reduction in the amount of total fatty acid (FA) and the area of triacylglycerol, increases in the ratios of saturated fatty acid and monounsaturated fatty acid (MUFA) to total FA in 5%-exposed *B*. *koreanus* were observed. Furthermore, mRNA expression analysis confirmed that CCR promoted the synthesis of MUFA through Δ9 desaturase. Moreover, expression of the docosahexaenoic acid (DHA) synthesizing gene Δ4 desaturase was also upregulated, together with DHA content. These data suggest that CCR modified protein acetylation and lipid metabolism, leading to a decrease in reproduction and consequently resulting in life span extension.

## Introduction

The energy trade-off between life span and reproduction is commonly observed in most animal taxa^1^. This phenomenon can be affected by various abiotic and biotic factors, such as temperature^2,3^, pH^4^, and metabolic changes^5^. Among them, chronic caloric restriction (CCR) is a well-known regulator leading to an energy trade-off in various organisms such as mice^6–8^, the grasshopper *Romalea microptera*^9^, the nematode *Caenorhabditis elegans*^10^, and the rotifer *Brachionus manjavacas*^11^. Based on previous findings, a simple hypothesis to explain this phenomenon is that this is one of the strategies organisms use to overcome unfavorable changes in their surrounding environment.

Many studies on the mechanisms of prolonging life span have focused on protein deacetylase. From yeast to human, life span is regulated by protein modification^12,13^. In particular, the gene encoding sirtuin, one of the protein deacetylases, is a well-known marker of life span regulation^14^. *Sirtuin* genes were initially identified as transcription-silencing protein deacetylases in yeast. Calorie restriction was shown to extend life span through upregulation of yeast sirtuin genes^15,16^. In addition, upregulation of *sirtuin2* was reported to extend life span in the fruit fly *Drosophila melanogaster*^17^, although there is still some controversy regarding this finding^18^. In mammals, seven *sirtuin* genes have been identified, and *sirtuin1* was found to have a similar function to *sirtuin2* in yeast in terms of longevity^19^. Therefore, study of the function of *sirtuin genes* is important in relation to life span.

In addition to protein deacetylase mechanisms, recent studies have revealed that CCR affects lipid metabolism. In mice, increasing the ratio of dietary saturated fatty acid (SFA) to monounsaturated fatty acid (MUFA) increases the life span by lowering the ratio of dietary polyunsaturated fatty acid (PUFA) composition^7^. Other researchers have found that CCR extends life span and affects overall lipid metabolism (e.g., circulating lipid, hepatic cholesterol level, triacylglycerol)^6^. In addition, an increase in life span was positively correlated with an increase in MUFA synthesis in H3K4me3 (trimethylates histone H3 on lysine 4) methyltransferase-deficient *C*. *elegans*^20^. However, since lipid metabolism plays a multifunctional role in various mechanisms such as long-term energy storage, intercellular and intracellular signaling, and membrane homeostasis, confirmation of the direct correlation between lipid metabolism and CCR remains a challenging issue.

As microzooplankton, rotifers (phylum Rotifera) are widely distributed throughout aquatic ecosystems and function as a bridge between producers and higher-level consumers in aquatic food chains^21^. Because of their small size (100–200 μm), easy maintenance, slow locomotion, and short reproductive cycle (~24 h), rotifers have established as an excellent experimental model organism in aquatic research^22,23^. Rotifers have also been used as a model species for aging experiments in response to temperature^24^, antioxidants^25^, and CCR^11,26,27^. In addition, a recent study used rotifers to screen drugs for follow-up in a vertebrate model^28^. Therefore, *B*. *koreanus* is a suitable organism to confirm the energy trade-off between lifespan and reproduction. Also, *B*. *koreanus* has the additional advantage over other aquatic species of the ability to reveal mechanisms as we have successfully identified its whole RNA sequence^29^.

The purpose of this investigation is to examine the interrelationship of aging extension and modification of lipid metabolism under CCR in *B*. *koreanus*. To assess the effect of CCR, we initially observed the life cycle parameters (life span, cumulative offspring, and offspring production per day) under eight different dietary conditions from *ad libitum* feeding with *T*. *suecica* (100%) to starvation (0%). Based on our results, we chose 5% *T*. *suecica*, which showed an increase in life span and decrease in fecundity, for further analysis of modulation of the expression of *sirtuin* and genes related to lipid metabolism and fatty acid composition under CCR in the rotifer *B*. *koreanus*. This study provides new insight into the interplay between CCR and the relationships between protein deacetylase genes, lipid metabolism, and life cycle parameters.

## Results

### Effects of CCR on Life Cycle Parameters

**Figure 1:**
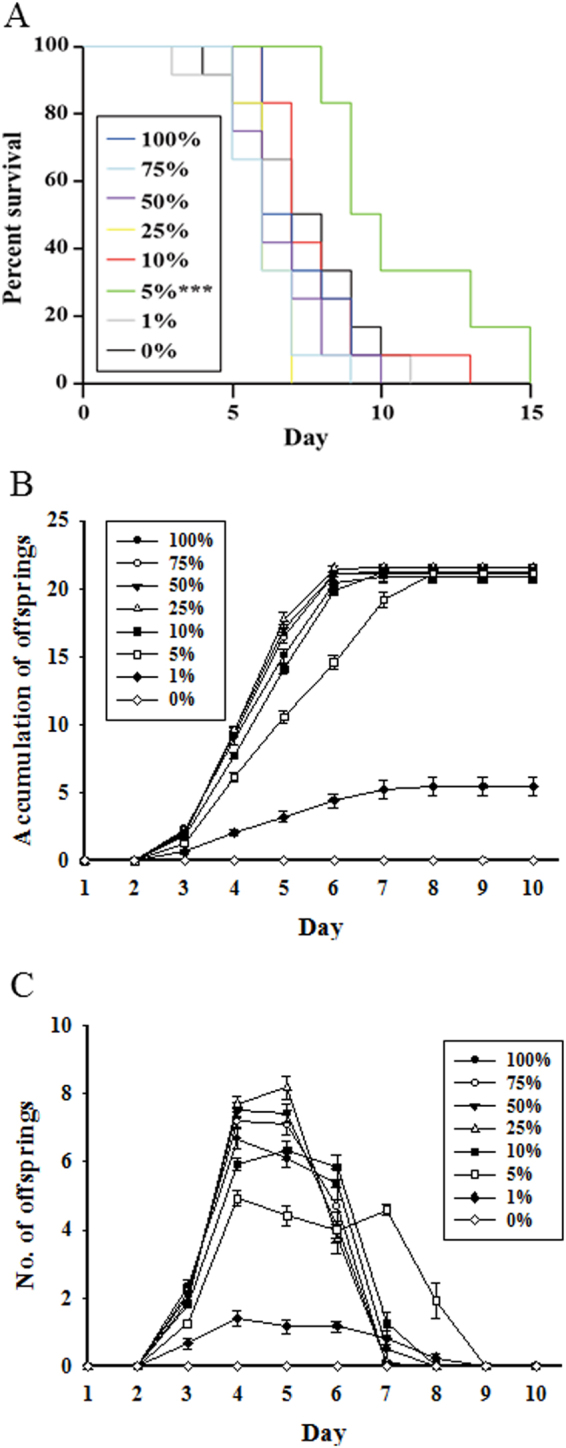
Effects of chronic caloric restriction on life cycle parameters in *B*. *koreanus* exposed to different concentrations of *T*. *suecica* (100 [Control], 75, 50, 25, 10, 5, 1, and 0%). (**A**) Life span, (**B**) cumulative offspring and (**C**) number of offspring per day. Asterisk (*) indicates significant difference between test and control groups (*P* < 0.05, Bonferroni’s correction).

Life cycle parameters were observed after exposure to different food concentrations (100, 75, 50, 25, 10, 5, 1, and 0%). In life span assessment (Fig. 1A and Suppl. Table 3), a significant (*P* < 0.05) extension of life span (about 48.6%) was observed only in the 5%-exposed group compared with the 100%-exposed group. Maximum and minimum life spans were 15 and 8 days in the 5%-exposed group and 10 and 6 days in the 100%-exposed group, respectively. In terms of cumulative offspring (Fig. 1B), 1%- and 5%-exposed groups showed a delay, and the 0% group did not produce any offspring. However, no changes in the mean total offspring were observed between the 100%- and 5%-exposed groups (Fig. 1B**;** Suppl. Table 3). Regarding the average number of offspring per day (Fig. 1C), CCR treatment resulted in a reduction in daily reproduction throughout the experiment, but the number of reproductive days was increased, especially in the 5%-exposed group. Based on *in-vivo* data, further experiments were performed using 100% being the control and 5% as test group.

### Modulation of *Sirtuin* Genes under CCR

**Figure 2:**
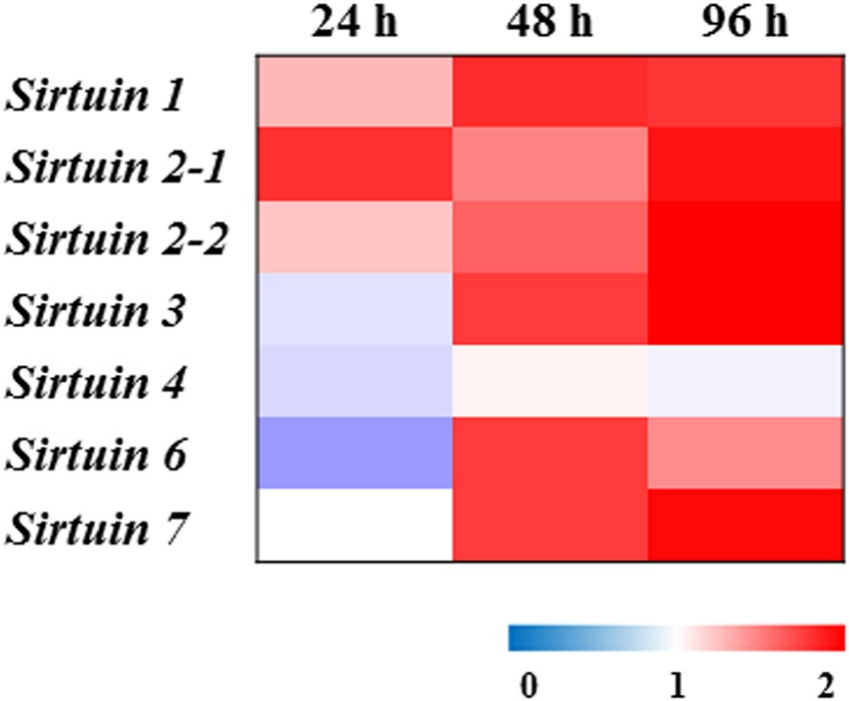
Transcription profile of sirtuin genes after exposure to 5% *T*. *suecica* at 24, 48, and 96 h represented as a heat map compared with the control (100%).

The mRNA expression of *sirtuin* genes of *B*. *koreanus* was measured under 5% *T*. *suecica* CCR conditions in a time-dependent manner (24, 48, and 96 h) (Fig. 2**;** Suppl. Table 2). With the exception of *sirtuin 4*, all of the *sirtuin* genes were significantly induced (*P* < 0.05) under CCR (5%-exposed group) compared with the control.

### The Area of Lipid Droplets in Response to CCR

**Figure 3:**
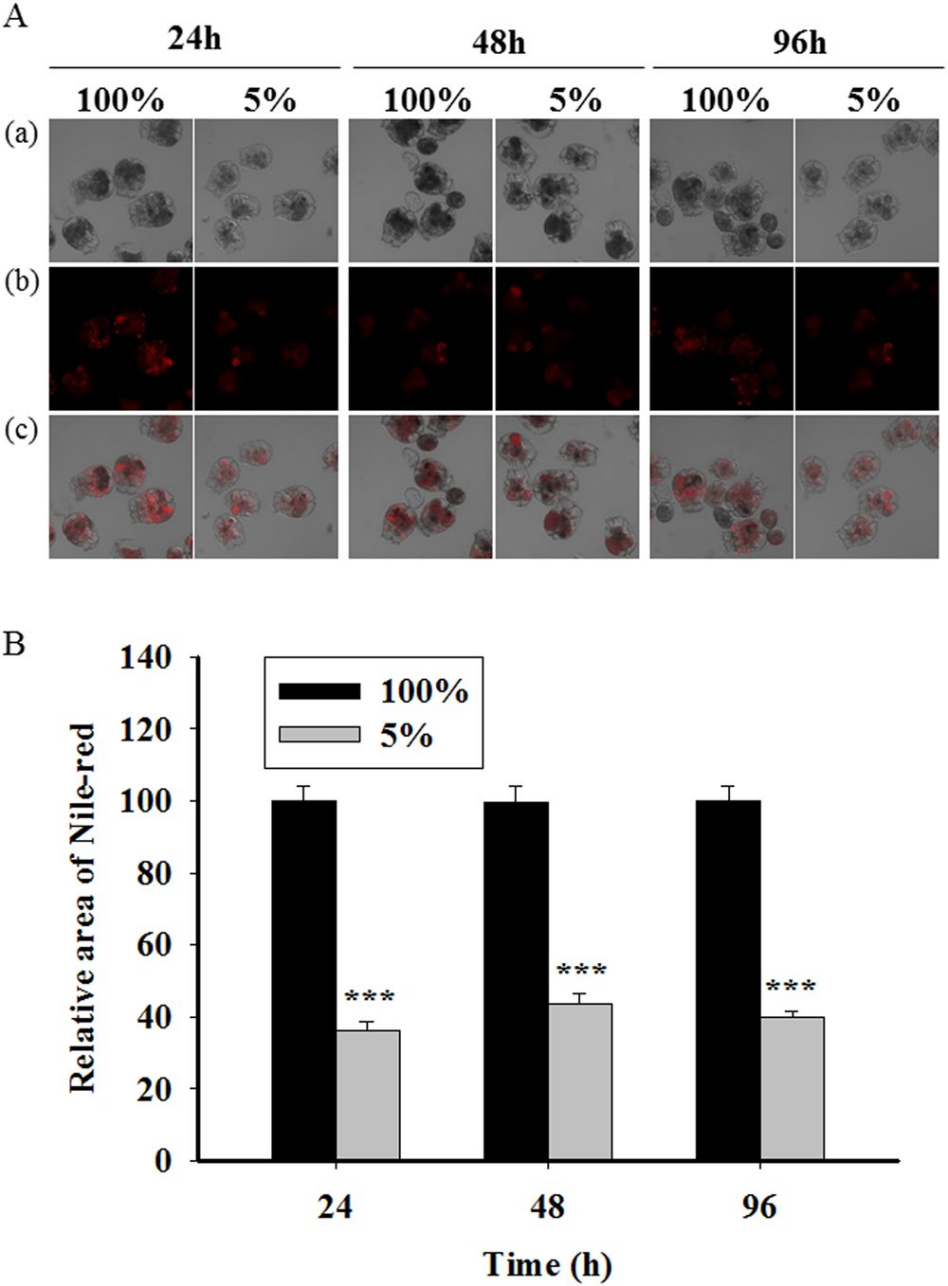
Lipid accumulation in *B*. *koreanus* exposed to 100% and 5% *T*. *suecica* for 24, 48, and 96 h. (**A**) Confocal microscopy analysis of Nile red-stained *B*. *koreanus*: (a) bright field, (b) fluorescent, and (c) merged. Red fluorescence indicates accumulated neutral lipids. (**B**) The area of triacylglycerol in *B*. *koreanus* is represented as a percentage of the relative area using the LAS image analysis tool (n = 30). Significant differences compared with the control value are indicated by an asterisk (*) (*P* < 0.05, Student’s *t*-test).

The area of LDs was measured after exposure to 100% and 5% *T*. *suecica* for 24, 48, and 96 h (Fig. 3). As expected, the area of the 5% group was significantly decreased (*P* < 0.05) compared with the control (100%) for all exposure times.

### Effect of CCR on Fatty Acid Composition

**Figure 4:**
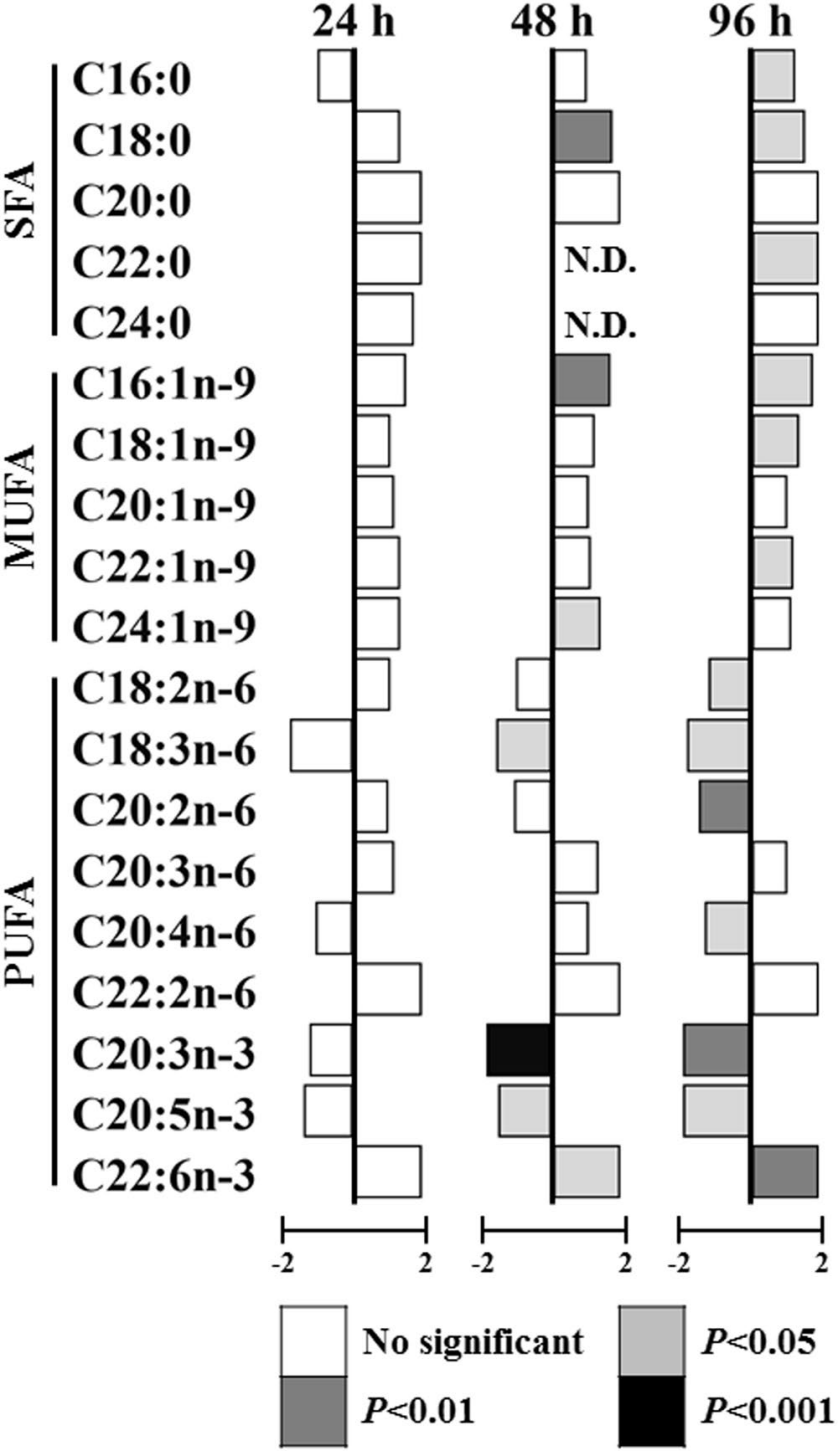
Effect of chronic caloric restriction on single fatty acid composition. Fatty acid profiles in the 5%-exposed group are represented as the difference in percentage abundance compared with the control (100%-exposed group). Statistical significances were determined by Student’s *t*-test (gray scale).

Fatty acid composition was measured after exposure to 100% and 5% *T*. *suecica* for 24, 48, and 96 h. Similar to the results for LDs area, the amount of total fatty acid was significantly decreased (*P* < 0.05) in the 5%-exposed group at all time points (Suppl. Table 4). Analysis of fatty acid composition in the 100% and 5% group revealed a decrease in most of the fatty acids (e.g., C18:1n-9, C20:3n-3, and C20:5n-3) in the 5%-exposed group. However, results for single fatty acids (Fig. 4) showed an increasing trend in the relative amounts of SFAs and MUFAs (*P* < 0.05). Regardless of the reduction in the relative amount of PUFA, a significant increase (*P* < 0.05) in the relative amount of DHA (C22:6n-3) was observed.

### Modulation of Genes Related to Lipid Metabolism under CCR

**Figure 5:**
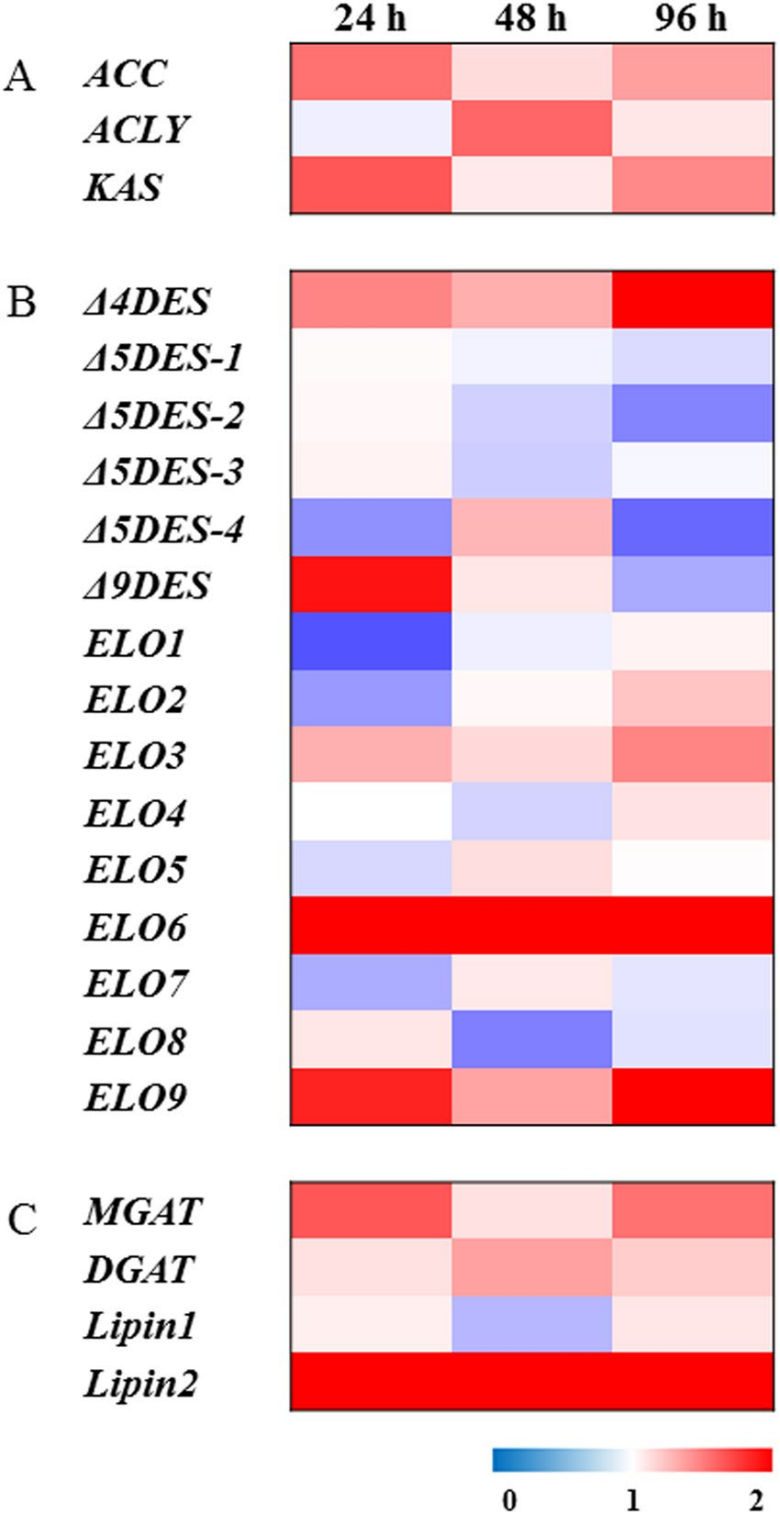
Transcription profile of lipid metabolism-related genes after exposure to 100% or 5% *T*. *suecica* at 24, 48, and 96 h represented by a heat map. (**A**) *De novo* lipogenesis genes (ATP-citrate lyase [*ACLY*], acetyl-CoA carboxylase [*ACC*], and β-keto-acyl-[acyl-carrier-protein] synthase [*KAS*]), (**B**) fatty acid structure modification genes (desaturase [*DES*] and elongase [*ELO*]), and (**C**) triacylglycerol formation genes (monoacylglycerol acyltransferase [*MGAT*], diacylglycerol acyltransferase [*DGAT*], and Lipin 1 and 2).

The mRNA expression of lipid metabolism-related genes was measured in the 5%-exposed group compared with the control (100%) at 24, 48, and 96 h (Fig. 5**;** Suppl. Table 2). Genes involved in *de novo* lipogenesis (DNL: acetyl-CoA carboxylase [*ACC*], ATP-citrate lyase [*ACLY*], and β-keto-acyl-[acyl-carrier-protein] synthase [*KAS*]) were significantly upregulated (*P* < 0.05) under CCR. Desaturases and elongases were also significantly modulated. Although food supply was lower in the 5%-exposed group than in the 100%-exposed group (control), Δ9 desaturase (at 24 h), Δ4 desaturase (at 96 h), and elongase 6 (at all time points) mRNAs were significantly upregulated (*P* < 0.05) compared with the control (100%).

## Discussion

Life span extension and reproductive retardation under CCR are common phenomena in most animals. However, studies on the interrelationship of the life cycle parameter outcomes and lipid metabolism under CCR over the entire lifetime are still insufficient. Therefore, in this study, we assessed life cycle parameters (e.g., life span and fecundity), measured the expression of protein deacetylase *sirtuin* genes and lipid metabolism–related genes, and examined fatty acid composition to investigate the aging extension and modification of lipid metabolism in the monogonont rotifer *B*. *koreanus* under CCR.

Among the various food concentrations tested (from 0% to 100% of *T*. *suecica*), only the 5%-exposed group demonstrated a delay in cumulative offspring and an increase in reproductive days with significant life span extension in *B*. *koreanus* (Fig. 1**;** Suppl. Table 3), indicating that life span extension and fecundity (cumulative offspring) under CCR showed an inverse relationship. This phenomenon, known as the disposable soma hypothesis, is also referred to as the “cost of reproduction” in rotifers^30,31^. Rotifers have been widely used to investigate the aging system^28,32^. For example, extension of the life span was demonstrated in a less than 25% treatment group compared to the 100% treatment group in *B*. *manjavacas*^11^. Also, in mass culture of the rotifer *B*. *plicatilis*, fasting after hatching (days 1–4) reduced the reproductive success rate but increased life span^33^. This phenomenon has been observed not only in the rotifer *Brachionus* species^11,33^, but also in mice^6–8^, grasshopper^9^, and nematode^10^. Taken together, these results are considered to be one of the strategy to reduce reproduction (cumulative offspring) and prolong the life span under conditions of insufficient food source.

We identified *sirtuin* genes, which are well-known protein deacetylases, in *B*. *koreanus* using RNA-seq.^29^ and a whole-genome database (GenBank no. PJRA00000000) produced in our laboratory and analyzed their mRNA expression under CCR. Based on the results of the life cycle parameters, the 5%-exposed group was suitable candidates for analysis of mRNA expression of sirtuin genes compared to the 100%-exposed group as the 5%-exposed group demonstrated an increase in mean life span. In *B*. *koreanus*, all sirtuin genes except sirtuin 4 were upregulated under CCR over 96 h (Fig. 2**;** Suppl. Table 2). In general, sirtuins are involved in cellular functions such as DNA repair, inflammatory response, cell cycle, and apoptosis^14^. In addition, recent studies have focused on the strong relationship between sirtuin expression and aging processes. For example, sirtuin 2 was first identified in yeast, and a correlation between sirtuin activity and longevity was revealed^16,34^. Furthermore, in mice, the expression of sirtuin genes increased life span^35,36^, whereas sirtuin knockout decreased life span^37,38^, indicating that expression of *sirtuin* genes is a key element in prolonging the life span. Furthermore, calorie restriction has been reported to extend life span through upregulation of sirtuins in yeast and mice^8,15,16^. To summarize, upregulation of *sirtuin* mRNA expression under CCR, which is similarly conserved from yeast to mammals, as well as in *B*. *koreanus*, implies the functional conservation of sirtuins as life span regulators.

Analysis of LDs and fatty acid composition showed that CCR modulated lipid metabolism in *B*. *koreanus* (Fig. 3**;** Suppl. Table 4). In our CCR model in the rotifer *B*. *koreanus*, the food exposure was only 5% of that of the control; therefore, as expected, the area of LDs and the absolute values of total and single fatty acids were decreased. Indeed, the changes in the LDs are likely commonly observed indication of the stressful condition in aquatic organisms. For example, under high temperature shift, trend between the survival of each generation and lipid contents was negatively correlated in the estuarine copepod *Eurytemora affinis*^39^. Furthermore, our results are concordant with findings in mice exposed to CCR, which showed a reduction in total body weight and decrease in TAG and cholesterol^7,8^. Despite the decrease in total fatty acid and the area of LDs, the ratio of SFA to MUFA among total fatty acids in the 5%-exposed group showed a relative increase in *B*. *koreanus* (Fig. 4). This result is consistent with the findings from mice and nematode. In mice^7^, the composition of dietary lipids has an effect on the life span. In particular, administration of an energy source containing a low ratio of PUFA and high ratio of MUFA and SFA resulted in life span extension. In addition, a knockout mutant of H3K4me3 methyltransferase, a life span prolonging gene, in the nematode *C*. *elegans*^12^ exhibited differences in the modulation of lipid metabolism in intestine and germline compared with the wild type^20^. For example, in the intestine, an increase in the synthesis of NL and MUFA and their synthesizing genes was observed, but levels of the germline target genes were reduced. Taken together, these data show that CCR reduces total fatty acid, the area of LDs, and reproduction, while promoting MUFA synthesis, which can further implicate to the extension of life span at the cost of the physiological changes.

The correlation between the expression of lipid metabolism-related genes and fatty acid composition was investigated in the rotifer *B*. *koreanus* under CCR (Fig. 5**;** Suppl. Table 2). DNL genes (*ACC*, *ACLY*, and *KAS*) are involved in the synthesis of fatty acids from glucose, the final metabolite of which is palmitic acid (PA, C16:0)^40^. In the present study, the amount of PA was initially decreased at 24 h but was increased significantly compared to the 100%-exposed group at 96 h, suggesting that increased expression of DNL genes led to the synthesis of PA from glucose. Also, the increase in PA might influence the proportion of MUFA in *B*. *koreanus*. The synthesis of MUFA is affected by an increase in Δ9 desaturase, which is the key gene involved in the production of MUFA (palmitoleic acid [16:1n-9] and oleic acid [C18:1n-9]) from saturated fatty acid (PA and stearic acid [C18:0])^41^. These results are consistent with findings in the H3K4me3 methyltransferase mutant of *C*. *elegans*, which showed life span extension and upregulation of *Δ9 desaturases*, leading to an increase in the MUFA content^12,20^.

An increase in Δ4 desaturase in the rotifer *B*. *koreanus* might have been involved in the synthesis of docosahexaenoic acid (DHA, C22:6n-3), which was the only increased PUFA, under CCR. In general, DHA has anti-inflammatory activity and is metabolized into inflammation-resolving mediators (e.g., resolvins, protectins, and maresins) through cyclooxygenase and lipoxygenase pathways^42,43^. There are two DHA synthetic pathways: a pathway created by β-oxidation after the action of elongase and Δ6 desaturase on docosapentaenoic acid (DPA, C22:5n-3) or direct synthesis of DHA from DPA using Δ4 desaturase^42^. We measured the composition of FA of *T*. *suecica* in normal condition, and interestingly, DHA was not detected (Suppl. Table 5). Other studies also reported very low DHA content in *T*. *suecica*^44^. Therefore, DHA in *B*. *koreanus* was only synthesized from other FA utilizing enzymes. Therefore, our results indicate that an increase in Δ4 desaturase expression modulates DHA synthesis. In terms of TAG synthesis in the rotifer *B*. *koreanus*, expression of *MGAT* and Lipin 2 genes was increased, which is consistent with the results of the H3K4me3 methyltransferase mutant *C*. *elegans*^20^. However, unlike the *C*. *elegans* experiment, the energy source administered to experimental organisms was limited to 5% of the control, thus the area of TAG appeared to decrease by approximately 60% compared with the control (100%).

To summarize the relationship between fatty acid composition and expression of lipid metabolism-related genes (Figs 4, 5**;** Suppl. Table 2), the mRNA expression level of lipid metabolism genes was analyzed. Based on our results, we suggest that the rotifer *B*. *koreanus* utilizes its energy to synthesize SFA and MUFA using the DNL pathway and Δ9 desaturase, respectively. Also, Δ4 desaturase is specifically involved in the synthesis of DHA, which is the only constituent of PUFA that is increased under CCR.

**Figure 6:**
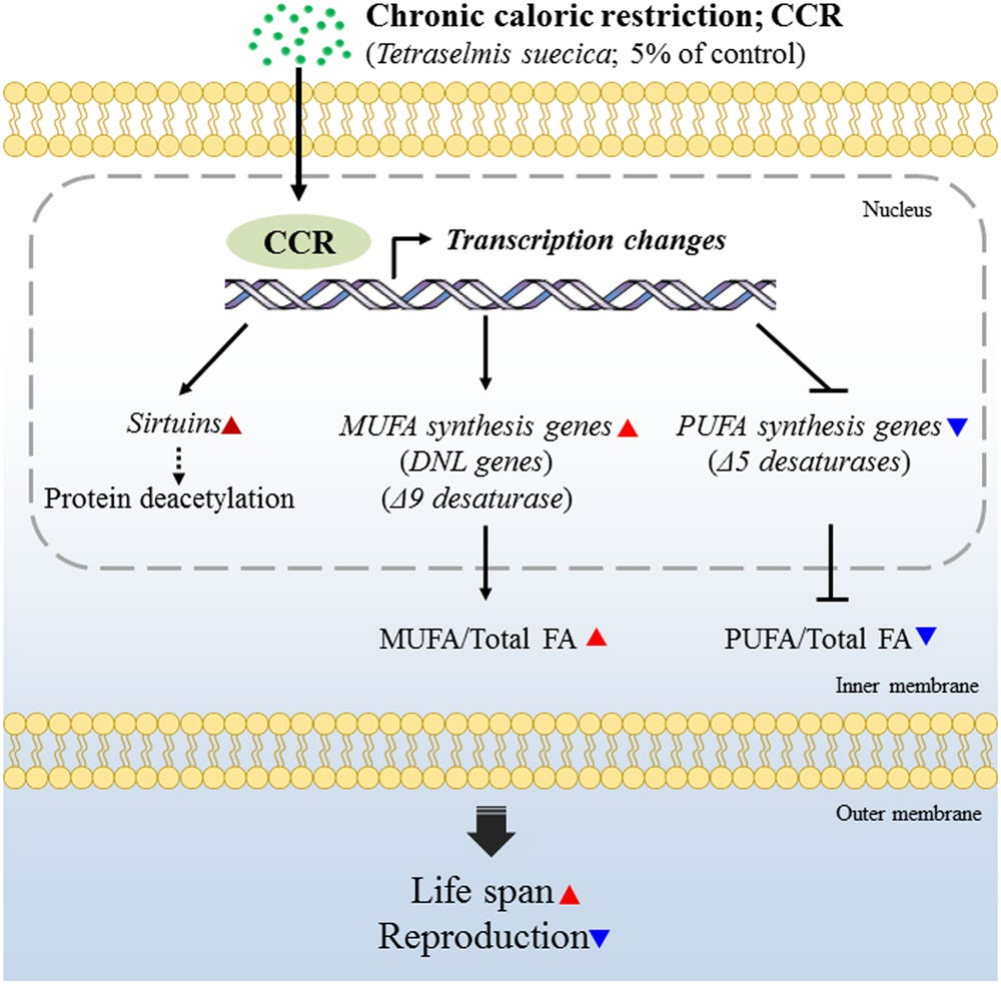
Schematic diagram of effect of chronic caloric restriction in the monogonont rotifer, *B*. *koreanus*. DNL genes: *De novo* lipogenesis genes; FA: Fatty acid; MUFA: monounsaturated fatty acid; PUFA: Polyunsaturated fatty acid.

In conclusion, the phenomenon called “cost of reproduction” was confirmed in the monogonont rotifer *B*. *koreanus*, via utilizing of aging extension mechanism under CCR through increase in mRNA expression (sirtuin and MUFA synthesis genes) and the proportional increase in MUFA (Fig. 6). From this investigation, it can be suggested that one of the survival strategies to endure food shortages is to modulate the mechanism (protein deacetylation and lipid metabolism) in *B*. *koreanus*, thereby lowering reproduction and increasing life span. This study will provide a valuable insight into the mechanism of aging extension and will contribute a step toward establishment of *B*. *koreanus* as a model species for in-depth mechanistical studies.

## Methods

### Culture and Assessment of Life Cycle Parameters

The species identification was confirmed by morphological analysis^45,46^ and sequencing of the mitochondrial DNA gene *CO1*^47^. In this study, one mL ASW with various concentrations of *T*. *suecica* (100 [6 × 10^5^ cells/ml], 75, 50, 25, 10, 5, 1, and 0%) were renewed every 24 h. We determined the number of *T*. *suecica* cells based on the reports on life-span extension by caloric restriction in the rotifer *B*. *manjavacas*^11,26^ and set 6 × 10^5^ cells/ml as 100% for this experiment. The detailed information on the procedure for the assessment of life cycle parameters is incorporated in the Supplementary file. All experiments were performed in biological triplicate.

### Measurement of Triacylglycerol in Response to CCR

To examine the effects of chronic caloric restriction on neutral lipid accumulation *in vivo*, Nile red staining was performed. Two groups of *B*. *koreanus* were fed 100% or 5% *T*. *suecica* for 24, 48, and 96 h. The 100% group was considered the control. A detailed Nile red staining method is provided in the Supplementary file.

### Analysis of Fatty Acid Composition under CCR

To analyze variations in fatty acid composition in response to chronic caloric restriction in *B*. *koreanus* (100% and 5% *T*. *suecica* at 24, 48, and 96 h), we followed the protocol provided by Hama and Handa^48^ with minor modifications, and detailed information is provided in the Supplementary section. All experiments were performed in triplicate.

### Expression of *Sirtuin* and Genes Related to Lipid Metabolism

To examine the expression of *sirtuin* and lipid metabolism-related genes, *in silico* analysis of *B*. *koreanus* RNA-seq information was performed^29^. The identification of *sirtuin* and lipid-metabolism-related genes were performed using BLAST analysis. To investigate the CCR-induced modulation of *sirtuin* and lipid metabolism-related genes, we measured mRNA expression levels over 96 h (24, 48, and 96 h) in response to 5% *T*. *suecica* exposure. All experiments were performed in technical triplicate. The information regarding identification of genes and the protocols for the mRNA expression are further provided in the Supplementary file.

### Statistical Analysis

For statistical analysis, SPSS ver. 18.0 (SPSS Inc., Chicago, IL, USA) was used and the data are presented as mean ± S.D. Student’s paired t-test and one-way ANOVA were used to analyze the significant differences between the control and test groups, followed by Tukey’s test. Using R statistical software (version 3.2.1 of the R Foundation for Statistical Computing Platform^©^, 2015), Kaplan–Meier survival curves for significance were calculated. Overall, the differences with *P* < 0.05 were considered significant.

## Electronic supplementary material

Supplementary information
