# Supplementary material for: Aging extension and modifications of lipid metabolism in the monogonont rotifer Brachionus koreanus under chronic caloric restriction
Source: Sci Rep. 2018 Jan 29;8:1741. doi: 10.1038/s41598-018-20108-7 (PMC5789037; doi:10.1038/s41598-018-20108-7)
Supplement: Supplementary file 1 — Supplementary information [file 41598_2018_20108_MOESM1_ESM.doc]

**Supplementary Information**

**Aging extension and modifications of lipid metabolism in the monogonont rotifer *Brachionus koreanus* under chronic caloric restriction**

**Min-Chul Lee^1^, Jun Chul Park^1^, Deok-Seo Yoon^1^, Jeonghoon Han^1^, Sujin Kang^2^, Shohei Kamizono^3^, Ae-Son Om^4^, Kyung-Hoon Shin^2^, Atsushi Hagiwara^3^ & Jae-Seong Lee^1,*^**

^1^Department of Biological Science, College of Science, Sungkyunkwan University, Suwon 16419, South Korea

^2^Department of Marine Sciences and Convergent Technology, Hanyang University, Ansan 15588, South Korea

^3^Graduate School of Fisheries and Environmental Sciences, Nagasaki University, Nagasaki 852-8521, Japan

^4^Department of Food and Nutrition, College of Human Ecology, Hanyang University, Seoul 04763, South Korea

____________________________

^*^Corresponding author: E-mail jslee2@skku.edu (J.-S. Lee)

**Table of Contents**

**I. SUPPLEMENTAL MATERIAL, METHODS**

**A. Supplemental material, Methods**∙∙∙∙∙∙∙∙∙∙∙∙∙∙∙∙∙∙∙∙∙∙∙∙∙∙∙∙∙∙∙∙∙∙∙∙∙∙∙∙∙∙∙∙∙∙∙∙∙∙∙∙∙∙∙∙∙∙∙∙∙∙∙∙∙∙∙∙∙∙∙∙∙∙∙∙∙**S3**

**B. References**∙∙∙∙∙∙∙∙∙∙∙∙∙∙∙∙∙∙∙∙∙∙∙∙∙∙∙∙∙∙∙∙∙∙∙∙∙∙∙∙∙∙∙∙∙∙∙∙∙∙∙∙∙∙∙∙∙∙∙∙∙∙∙∙∙∙∙∙∙∙∙∙∙∙∙∙∙∙∙∙∙∙∙∙∙∙∙∙∙∙∙∙∙∙∙∙∙∙∙∙∙∙∙∙∙∙∙∙∙∙∙∙∙∙**S5**

**II. SUPPLEMENTAL TABLE**

**Supplementary Table 1** GenBank accession numbers and primer sets used in this study∙∙∙∙∙∙∙∙∙∙∙∙∙∙∙∙∙∙∙∙∙∙∙∙∙∙∙∙∙∙∙∙∙∙∙∙∙∙∙∙∙∙∙∙∙∙∙∙∙∙∙∙∙∙∙∙∙∙∙∙∙∙∙∙∙∙∙∙∙∙∙∙∙∙∙∙∙∙∙∙∙∙∙∙∙**S7**

**Supplementary Table 2** Relative value of mRNA expression after exposure to 100% and 5% *T. suecica* for 24, 48, and 96 h∙∙∙∙∙∙∙∙∙∙∙∙∙∙∙∙∙∙∙∙∙∙∙∙∙∙∙∙∙∙∙∙**S9**

**Supplementary Table 3** Life span and the mean total offspring under different food concentrations∙∙∙∙∙∙∙∙∙∙∙∙∙∙∙∙∙∙∙∙∙∙∙∙∙∙∙∙∙∙∙∙∙∙∙∙∙∙∙∙∙∙∙∙∙∙∙∙∙∙∙∙∙∙∙∙∙∙∙∙∙∙∙∙∙∙∙∙**S10**

**Supplementary Table 4** Composition of fatty acids after exposure to 100% and 5% *T. suecica* for 24, 48, and 96 h∙∙∙∙∙∙∙∙∙∙∙∙∙∙∙∙∙∙∙∙∙∙∙∙∙∙∙∙∙∙∙∙∙∙∙∙∙∙∙∙∙∙∙∙∙∙∙**S11**

**Supplementary Table 5** Composition of fatty acids of *Tetraselmis suecica*∙∙∙∙∙∙∙∙∙∙∙∙∙∙∙**S12**

**I. SUPPLEMENTAL MATERIAL, METHODS**

**A. Supplemental material, Methods**

**Culture and Maintenance of *Brachionus koreanus***

The rotifer *B. koreanus* was collected from a hatchery of East Sea Fisheries Research Institute, Uljin (36º58’43.01”N, 129º24’28.40”E) and maintained in the Department of Biological Science, Sungkyunkwan University, Suwon, South Korea, for use in this study. *B. koreanus* was fed the green marine microalgae *Tetraselmis suecica* (6×10^5^ cells/ml) every 24 h and maintained in 15 ppt filtered artificial seawater (ASW) (TetraMarine Salt Pro, Tetra™, Cincinnati, OH, USA) under laboratory conditions of 12:12 h (light:dark) photoperiod at 25°C. Species identification was confirmed by morphological analysis^1,2^ and sequencing of the mitochondrial DNA gene *CO1*^3^.

**Assessment of Life Cycle Parameters**

To examine life cycle parameters of *B. koreanus*, which reproduce asexually, we recorded life span, cumulative offspring, and number of offspring per day. The experiment was performed using the offspring (< 2 h after hatching), and newborn *B. koreanus* were transferred into wells of a new 24-well culture plate (SPL Life Science Co. Ltd., Seoul, South Korea) containing one mL ASW with various concentrations of *T. suecica* (100 [6×10^5^ cells/ml], 75, 50, 25, 10, 5, 1, and 0%) every 24 h. We determined the number of *T. suecica* cells based on the reports on life-span extension by caloric restriction in the rotifer *Brachionus manjavacas*^4,5^ and set 6×10^5^ cells/ml as 100% in this experiment. Stereomicroscopy (M205-A, Leica Microsystems, Wetzlar, Germany) was used to observe *B. koreanus.* The number of newborn rotifers was counted every 12 h to examine offspring production, while dead rotifers were counted to quantify the life span. All experiments were performed in biological triplicate.

**Measurement of Triacylglycerol in Response to CCR**

To examine the effects of chronic caloric restriction on neutral lipid accumulation *in vivo*, Nile red staining was performed. Two groups of *B. koreanus* were fed 100% or 5% *T. suecica* for 24, 48, and 96 h. The 100% group was considered the control. Nile red (1 mg/mL in acetone) was prepared immediately before use. *B. koreanus* was placed in formaldehyde (4%) for fixation and stained using Nile red (final concentration 2.5 μg/mL) for 5 min. Fixed and stained *B. koreanus* were viewed under a confocal laser scanning microscope (LSM 510 META; Zeiss, Oberkochen, Germany) at 543 nm excitation and 560-615 nm emission wavelengths. Neutral lipids were observed as red fluorescent bodies, and the accumulation of LDs in response to CCR was compared with that of the control (100%) for each time period. The area of LDs was analyzed with an LAS image analysis tool (ver. 4.3; Leica). For each test group, 30 rotifers were used and incubated in 50 mL ASW with *T. suecica*. All experiments were performed in triplicate.

**Analysis of Fatty Acid Composition under CCR**

To analyze variations in fatty acid composition in response to chronic caloric restriction in *B. koreanus*, we followed the protocol provided by Hama and Handa^6^ with minor modifications. Briefly, the lipids from CCR conditions (100% and 5% *T. suecica* at 24, 48, and 96 h) were extracted with dichloromethane/methanol 2:1 (v/v). Nonadecanoic acid (C19:0) was added to the extracts as an internal standard. Extraction procedures were repeated three times with sonication. The lipid fraction was separated from the water-methanol phase and converted into fatty acid methyl esters (FAMEs) by saponification using 0.5 M KOH-methanol, followed by methylation with BF3-methanol. Concentrations and compositions of FAMEs formed were analyzed in a gas chromatograph (GC-2010, Shimadzu, Kyoto, Japan) with a flame ionization detector using a fused silica capillary column (DB-5, 30 m × 0.25 mm i.d., 0.25 μm film thickness). Helium was used as a carrier gas. Samples were injected in splitless mode at an initial oven temperature of 40°C, which was increased to 200°C at 10°C/min and finally to 300°C at 2°C/min. FAs were identified from the retention times (RTs) of standards and mass spectra from gas chromatograph-mass spectrometry (GCMS-QP2010 Plus; Shimadzu, Kyoto, Japan). All experiments were performed in triplicate.

**Expression of *Sirtuin* and Genes Related to Lipid Metabolism**

To examine the expression of *sirtuin* and lipid metabolism-related genes, *in silico* analysis of *B. koreanus* RNA-seq information was performed^7^. Genes were subjected to BLAST analysis in the GenBank non-redundant (NR; including all GenBank, EMBL, DDBJ, and PDB sequences except EST, STS, GSS, and HTGS) amino acid sequence database (<http://blast.ncbi.nlm.nih.gov/>). The amplicons were sequenced on an ABI PRISM 3700 DNA analyzer, and putative transcription factor binding sites were screened using Geneious (v.10.0.7; Biomatters Ltd, Auckland, New Zealand)^8^. To investigate the CCR-induced modulation of *sirtuin* and lipid metabolism-related genes, we measured mRNA expression levels over 96 h (24, 48, and 96 h) in response to 5% *T. suecica* exposure. Total RNAs were extracted with TRIZOL® reagent (Invitrogen, Paisley, Scotland, UK) according to the manufacturer’s instructions. The quantity and purity were analyzed spectrometrically at 230, 260, and 280 nm (QIAxpert, Qiagen, Hilden, Germany). To synthesize cDNA for quantitative real-time reverse transcription-polymerase chain reaction (qRT-PCR), two µg of total RNA and oligo(dT)_20_ primer were used for reverse transcription (SuperScript™ II RT kit, Invitrogen, Carlsbad, CA, USA). qRT-PCR was conducted under the following conditions: 95°C/4 min; 40 cycles of 95°C/30 s, 55°C/30 s, 72°C/30 s, and 72°C/10 min using SYBR Green as a probe (Molecular Probes Inc., Eugene, OR, USA) in a CFX96^TM^ real-time PCR system (Bio-Rad, Hercules, CA, USA). To confirm the amplification of specific products, melting curve cycles were run at the following conditions: 95°C/1 min; 55°C/1 min; and 80 cycles of 55°C/10 s with a 0.5°C increase per cycle using qRT-PCR F or R primers **(Suppl. Table 1)**. The *B. koreanus* elongation factor 1-alpha (*EF1-α*) gene, which showed stable expression throughout the experiments, was used as an internal control to normalize expression levels between samples. All experiments were performed in technical triplicate. The relative fold-change in gene expression compared to the control was calculated by the 2^−ΔΔC^_T_ comparative method^9^.

**B. Reference**

1. Hwang, D.-S., Dahms, H.-U., Park, H.G. & Lee, J.-S. A new intertidal *Brachionus* and intrageneric phylogenetic relationships among *Brachionus* as revealed by allometry and *CO1-ITS1* gene analysis. *Zool. Stud.* **52**, 13 (2013).

2. Mills, S. *et al*. Fifteen species in one: deciphering the *Brachionus plicatilis* species complex (Rotifera, Monogononta) through DNA taxonomy. *Hydrobiologia* **796**, 39–58 (2017).

3. Hwang, D.-S. *et al*. Complete mitochondrial genome of the monogonont rotifer, *Brachionus koreanus* (Rotifera, Brachionidae). *Mito. DNA* **25**, 29–30 (2014).

4. Gribble, K.E. & Mark Welch, D.B. Life-span extension by caloric restriction is determined by type and level of food reduction and by reproductive mode in *Brachionus manjavacas* (Rotifera). *J. Gerontol. A Biol. Sci. Med. Sci.* **68**, 349–358 (2012).

5. Gribble, K.E., Jarvis, G., Bock, M. & Mark Welch, D.B. Maternal caloric restriction partially rescues the deleterious effects of advanced maternal age on offspring. *Aging Cell* **13**, 623–630 (2014).

6. Hama, T. & Handa N. Pattern of organic matter production by natural phytoplankton population in a eutrophic lake. I. Intracellular products. *Arch. Hydrobiol.* **109**, 107–120 (1987).

7. Lee, B.-Y. *et al*. Whole transcriptome analysis of the monogonont rotifer *Brachionus koreanus* provides molecular resources for developing biomarkers of carbohydrate metabolism. *Comp. Biochem. Physiol. D* **14**, 33–41 (2015).

8. Kearse, M. *et al*. Geneious basic: an integrated and extendable desktop software platform for the organization and analysis of sequence data. *Bioinformatics* **28**, 1647–1649 (2012).

9. Livak, K.J. & Schmittgen, T.D. Analysis of relative gene expression data using real-time quantitative PCR and the 2^−ΔΔC^_T_ method. *Methods* **25**, 402–408 (2001).

**II. SUPPLEMENTAL TABLE**

**Supplementary Table 1 GenBank accession numbers and primer sets used in this study**

| **Gene** | **GenBank**  **Accession No.** | **Oligo name** | **Sequence (5′ -> 3′)** |
| --- | --- | --- | --- |
| *ATP-citrate lyase*  *(ACLY)* | MF768956 | F | CCAGTTCCTCCAACTGTTCC |
|  |  | R | CAGCATATAAAAGCTCTTGGC |
| *Acetyl-CoA carboxylase*  *(ACC)* | MF768955 | F | GAAAGCGCTAAGAGCTCAGTG |
|  |  | R | CCGATATTTGATGATAAATGGG |
| *Beta-keto-acyl-synthase*  *(KAS)* | MF768976 | F | GAGTCCAAATGGAAACGGTG |
|  |  | R | CACTTATTTCAGCAATGTCACC |
| *Elongase 1* | MF768965 | F | CATCATTCGACCATGTTGATGA |
|  |  | R | GCTTGGAATAACTGAAAAAGCG |
| *Elongase 2* | MF768966 | F | TGTGTGGCCAGAATTTGTCTAT |
|  |  | R | AATATAAACAGCCATGACCAGC |
| *Elongase 3* | MF768967 | F | GCCATGAGAATGGTTTCAGTTT |
|  |  | R | AGATGATGGAAAACATGCAGAA |
| *Elongase 4* | MF768968 | F | TTTTGGGTACAATCAGAACGTG |
|  |  | R | TGAACATAAATGCCCAGAAACC |
| *Elongase 5* | MF768969 | F | TAAGGCAAGAGATTTTGGTTTGT |
|  |  | R | ATCCCTTAGTAATGTAAAACCACC |
| *Elongase 6* | MF768970 | F | CTGGTTGCTGGACATATTTGTT |
|  |  | R | AAATAAGAACAGTGGCATGGTG |
| *Elongase 7* | MF768971 | F | CAATTTTTGCCAGGTCATAGGA |
|  |  | R | CTAAGCATTCGTATGGCAAGAG |
| *Elongase 8* | MF768972 | F | AATGATTGTAACAGGATGGCTC |
|  |  | R | GATGGACCCATTGAAGACAATC |
| *Elongase 9* | MF768973 | F | GCCAGGTTATCGCTTCATTTTA |
|  |  | R | TTTTGGCTAAATGGTTTTCGGA |
| *Δ4 desaturase* | MF768957 | F | GGAGGCTACCTGAAGTTAGAAA |
|  |  | R | GAATAAGGACCCATTTCAGTGC |
| *Δ5 desaturase-1* | MF768959 | F | AATAAAATATGGTGGTGGCTGG |
|  |  | R | GTTCCATAGAAGAGACGAGTGA |
| *Δ5 desaturase-2* | MF768961 | F | TTCACAAGCTTTCATAACGACA |
|  |  | R | TGAAAATCCTTATGCAACTCGG |
| *Δ5 desaturase-3* | MF768962 | F | TTTTTCACTTTGCCTCCATCTT |
|  |  | R | ACTGAAAATTCCGATCCACATT |
| *Δ5 desaturase-4* | MF768963 | F | TGACAAGGAAATTGCGATTAGG |
|  |  | R | AAGACAATGTGAAATCCATGCA |
| *Δ9 desaturase* | MF768964 | F | TGTGCTTCTATGCCAAAATTCA |
|  |  | R | TGGCTTTGTAAGTCCTATGTGA |
| *Monoacylglycerol acyltransferase* (*MGAT*) | MF768975 | F | AGTATGTGCACTGTTAATTGGA |
|  |  | R | GTGCTCCTGTTTCTAAAGCTAT |
| *Diacylglycerol acyltransferase* (*DGAT*) | MF768974 | F | TGGCCTTCATTACTAGCTTTAG |
|  |  | R | ATGAAACAGAAAAATTTCCCGC |
| *Lipin1* | KY828433 | F | CATTTTATGTGCGCTTTGGAAA |
|  |  | R | ACCAGATTCGTCTAGCATCATT |
| *Lipin2* | KY828434 | F | AGTGACGATGAAGATGAGGAAT |
|  |  | R | CTTTTCGACTATTCGGTGACCA |
| *SIRT1* | MF945612 | F | ATTACTAGGAGATTGTGATG |
|  |  | R | GGTCTTCTATTTGATTTAAA |
| *SIRT2-1* | MF945613 | F | GTTATGGGAACTAGTTTAAA |
|  |  | R | CAAGACGATAATTTTTATCT |
| *SIRT2-2* | MF945614 | F | GATTTTAGCAGATGTAAATT |
|  |  | R | CTATTGATTAAAGCTCTAGG |
| *SIRT3* | MF945615 | F | GTTAAAACTTTTATGAGAGC |
|  |  | R | GATAAATTGCTCTTATCATT |
| *SIRT4* | MF945616 | F | GGAAGTCTATTCAGCTTATA |
|  |  | R | AACTTTGCTACAGAGTGTAT |
| *SIRT6* | MF945617 | F | TAGTGATAATAAATTTGCAA |
|  |  | R | CTAATTCTAATTTCTCTTGG |
| *SIRT7* | MF945618 | F | TGTAACAAATTATGAAGTCA |
|  |  | R | TTAGTTTCTATTGGATTTTT |
| *EF1-α* | KY828435 | F | CAAGAAGCTGCCATCATC |
|  |  | R | CAGTTTGTCTCATATCACGAAC |

|  | 24 h | | 48 h | | 96 h | |
| --- | --- | --- | --- | --- | --- | --- |
|  | 100% | 5% | 100% | 5% | 100% | 5% |
| *Sirtuin1* | 1.00±0.03 | 1.28±0.09 | 1.00±0.02 | 1.83±0.04*** | 1.00±0.04 | 1.79±0.08*** |
| *Sirtuin2-1* | 1.00±0.05 | 1.81±0.24** | 1.00±0.09 | 1.49±0.08** | 1.00±0.03 | 1.92±0.10*** |
| *Sirtuin2-2* | 1.00±0.06 | 1.23±0.02 | 1.00±0.06 | 1.62±0.16** | 1.01±0.14 | 2.15±0.12*** |
| *Sirtuin3* | 1.00±0.10 | 0.89±0.07 | 1.01±0.15 | 1.77±0.40* | 1.00±0.08 | 2.22±0.08*** |
| *Sirtuin4* | 1.00±0.08 | 0.85±0.06 | 1.00±0.05 | 1.05±0.05 | 1.00±0.08 | 0.95±0.10 |
| *Sirtuin6* | 1.00±0.05 | 0.61±0.04*** | 1.01±0.15 | 1.77±0.16** | 1.00±0.08 | 1.45±0.09** |
| *Sirtuin7* | 1.01±0.18 | 1.00±0.28 | 1.00±0.02 | 1.77±0.25* | 1.02±0.24 | 1.97±0.21* |
| *ACC* | 1.00±0.02 | 1.56±0.03*** | 1.00±0.03 | 1.14±0.07 | 1.00±0.03 | 1.38±0.03*** |
| *ACLY* | 1.00±0.09 | 0.94±0.12 | 1.02±0.23 | 1.60±0.30 | 1.00±0.05 | 1.10±0.14 |
| *KAS* | 1.00±0.07 | 1.66±0.08*** | 1.00±0.04 | 1.09±0.05 | 1.00±0.05 | 1.47±0.14* |
| *Δ4DES* | 1.00±0.05 | 1.48±0.08** | 1.01±0.18 | 1.32±0.23 | 1.00±0.07 | 3.88±0.53** |
| *Δ5DES-1* | 1.00±0.12 | 1.02±0.04 | 1.00±0.12 | 0.95±0.10 | 1.00±0.05 | 0.86±0.07 |
| *Δ5DES-2* | 1.00±0.07 | 1.03±0.04 | 1.00±0.05 | 0.82±0.09* | 1.00±0.03 | 0.52±0.08** |
| *Δ5DES-3* | 1.00±0.09 | 1.05±0.09 | 1.01±0.17 | 0.81±0.09 | 1.01±0.22 | 0.97±0.07 |
| *Δ5DES-4* | 1.00±0.08 | 0.57±0.01** | 1.00±0.10 | 1.29±0.16 | 1.01±0.14 | 0.41±0.04** |
| *Δ9DES* | 1.00±0.05 | 1.93±0.16* | 1.00±0.09 | 1.10±0.02 | 1.00±0.09 | 0.67±0.03** |
| *ELO1* | 1.00±0.06 | 0.33±0.04*** | 1.00±0.09 | 0.94±0.04 | 1.01±0.13 | 1.05±0.08 |
| *ELO2* | 1.00±0.07 | 0.60±0.05** | 1.00±0.11 | 1.03±0.04 | 1.02±0.27 | 1.23±0.14 |
| *ELO3* | 1.00±0.11 | 1.31±0.06 | 1.00±0.01 | 1.15±0.27 | 1.00±0.05 | 1.48±0.31 |
| *ELO4* | 1.00±0.08 | 1.00±0.12 | 1.01±0.14 | 0.83±0.08 | 1.00±0.06 | 1.11±0.07 |
| *ELO5* | 1.00±0.06 | 0.85±0.02 | 1.01±0.20 | 1.13±0.06 | 1.01±0.2 | 1.01±0.02 |
| *ELO6* | 1.00±0.07 | 2.26±0.15*** | 1.00±0.12 | 2.76±0.08*** | 1.00±0.08 | 3.74±0.44*** |
| *ELO7* | 1.00±0.02 | 0.68±0.20 | 1.00±0.10 | 1.09±0.08 | 1.00±0.00 | 0.90±0.09 |
| *ELO8* | 1.00±0.01 | 1.10±0.25 | 1.00±0.05 | 0.50±0.03*** | 1.00±0.08 | 0.89±0.07 |
| *ELO9* | 1.00±0.08 | 1.87±0.07*** | 1.00±0.05 | 1.36±0.03** | 1.01±0.14 | 2.00±0.11** |
| *MGAT* | 1.00±0.02 | 1.66±0.03*** | 1.00±0.01 | 1.12±0.06* | 1.01±0.18 | 1.56±0.07* |
| *DGAT* | 1.00±0.02 | 1.12±0.14 | 1.03±0.28 | 1.37±0.19 | 1.01±0.13 | 1.20±0.13 |
| *Lipin1* | 1.00±0.01 | 1.06±0.02 | 1.00±0.03 | 0.72±0.04** | 1.00±0.02 | 1.10±0.13 |
| *Lipin2* | 1.00±0.03 | 5.49±0.80* | 1.00±0.04 | 3.63±0.81** | 1.00±0.05 | 4.70±0.42** |

**Supplementary Table 2** Relative value of mRNA expression after exposure to 100% and 5% *T. suecica* for 24, 48, and 96 h. Values are mean±SEM. Asterisk (*) indicates a significant difference between groups (**P*<0.05; ***P*<0.01; ****P*<0.001, Student’s *t*-test).

**Supplementary Table 3** Life span and mean total offspring under different food concentrations. Values are mean±SEM. Asterisk (*) indicates a significant difference between groups (**P*<0.05; ***P*<0.01; ****P*<0.001, Student’s *t*-test).

| Food concentration  (% ad libitum) | Minimum  life span (days) | Mean life span  (days; SEM) | Maximum  life span (days) | Mean total offspring  (days; SEM) |
| --- | --- | --- | --- | --- |
| 100 | 6 | 7.2 (1.4) | 10 | 20.9 (1.8) |
| 75 | 5.5 | 6.5 (1.1) | 9 | 21.1 (2) |
| 50 | 5.5 | 6.7 (1.4) | 10 | 21.2 (2.1) |
| 25 | 5.5 | 6.3 (0.6) | 7 | 21.6 (0.8) |
| 10 | 6.5 | 7.9 (1.8) | 13 | 21.2 (1.5) |
| 5 | 8 | 10.7 (2.6)*** | 15 | 21.1 (1.6) |
| 1 | 3 | 7.3 (2) | 11 | 5.5 (2.4)*** |
| 0 | 4.5 | 7.5 (2) | 11 | 0 (0)*** |

**Supplementary Table 4 Composition of fatty acids after exposure to 100% and 5% *T. suecica* for 24, 48, and 96 h. Values are mean±SEM. Asterisk (*) indicates a significant difference between groups (**P*<0.05; ***P*<0.01; ****P*<0.001, Student’s *t*-test).**

|  | Type of fatty acid  (µg/mg dry weight) | 24 h | | 48 h | | 96 h | |
| --- | --- | --- | --- | --- | --- | --- | --- |
|  |  | 100% | 5% | 100% | 5% | 100% | 5% |
| SFA | C16:0 | 11.07±2.34 | 6.41±2.15^*^ | 11.41±2.64 | 4.98±1.28^**^ | 10.42±1.57 | 6.67±1.05^*^ |
|  | C18:0 | 1.72±0.15 | 1.42±0.33 | 2.08±0.44 | 1.59±0.51 | 2.3±0.71 | 1.74±0.35 |
|  | C20:0 | 0.04±0.03 | 0.11±0.14 | 0.07±0.01 | 0.02±0.05 | 0.07±0.03 | 0.14±0.1 |
|  | C22:0 | 0.03±0.02 | 0.02±0.04 | 0.04±0.01 | N.D. | 0.04±0.02 | 0.04±0.04 |
|  | C24:0 | 0.04±0.03 | 0.02±0.04 | 0.05±0.01 | N.D. | 0.04±0.01 | 0.18±0.21 |
| ω9 | C16:1 | 0.61±0.17 | 0.54±0.21 | 0.8±0.18 | 0.6±0.18 | 0.79±0.11 | 0.72±0.2 |
|  | C18:1 | 3.47±0.43 | 2.27±0.71^*^ | 4.34±1.51 | 2.23±0.47^*^ | 4.62±0.68 | 3.14±0.47^*^ |
|  | C20:1 | 2.32±0.45 | 1.62±0.54 | 2.66±0.79 | 1.16±0.24^*^ | 2.76±0.57 | 1.43±0.13^*^ |
|  | C22:1 | 0.86±0.07 | 0.48±0.38 | 1.07±0.19 | 0.52±0.09^**^ | 1.16±0.15 | 0.69±0.09^*^ |
|  | C24:1 | 0.38±0.04 | 0.32±0.1 | 0.48±0.06 | 0.28±0.04^**^ | 0.51±0.05 | 0.19±0.17 |
| ω6 | C18:2 | 5.49±1.3 | 3.55±1.25 | 7.84±1.51 | 3.34±0.92^**^ | 8.83±1.11 | 3.61±0.22^**^ |
|  | C18:3 | 0.56±0.35 | 0.17±0.05 | 0.62±0.32 | 0.16±0.06^*^ | 0.72±0.09 | 0.19±0.04^**^ |
|  | C20:2 | 0.28±0.08 | 0.17±0.06 | 0.41±0.11 | 0.16±0.04^**^ | 0.51±0.03 | 0.17±0.02^***^ |
|  | C20:3 | 0.49±0.18 | 0.33±0.13 | 0.58±0.2 | 0.28±0.2 | 0.63±0.08 | 0.32±0^**^ |
|  | C20:4 | 1.21±0.51 | 0.65±0.35 | 1.52±0.82 | 0.66±0.22 | 1.63±0.1 | 0.59±0.04^**^ |
|  | C22:2 | 0.04±0.03 | 0.31±0.51 | 0.06±0.01 | 0.05±0.06 | 0.07±0.03 | 0.05±0.05 |
| ω3 | C20:3 | 1.09±0.33 | 0.51±0.23^*^ | 1.51±0.45 | 0.35±0.14^**^ | 1.92±0.16 | 0.44±0.01^**^ |
|  | C20:5 | 6.2±2.84 | 2.6±1.35 | 7.78±3.34 | 2.16±0.81^*^ | 8.28±1.43 | 1.98±0.24^*^ |
|  | C22:6 | 0.34±0.5 | 0.31±0.4 | 0.12±0.09 | 0.2±0.23 | 0.11±0.03 | 0.11±0.1 |
| Total | | 36.24±8.64 | 21.81±7.40^***^ | 43.45±12.37 | 18.74±4.78^***^ | 45.39±5.94 | 22.39±1.87^***^ |

**Supplementary Table 5. Composition of fatty acids of *Tetraselmis suecica*. Values are mean±SEM.**

| Type of fatty acid | Content (ng/μg) |
| --- | --- |
| C14:0 | 0.13±0.02 |
| C15:0 | 0.03±0 |
| C16:1 | 0.12±0.01 |
| C16:0 | 8.26±1.41 |
| C17:0 | 0.01±0 |
| C18:3ω6 | 0.07±0.02 |
| C18:2ω6c | 0.59±0.1 |
| C18:1ω9t | 0.89±0.15 |
| C18:0 | 0.35±0.14 |
| C20:4ω6 | 0.14±0.04 |
| C20:5ω3 | 0.49±0.13 |
| C20:3ω3 | 0.48±0.09 |
| C22:6ω3 | 0±0 |
| C22:1ω9 | 0±0 |
| C24:0 | 0.01±0 |
